# An Audit of the Technical Quality and Iatrogenic Errors of Root Canal Treatment by Undergraduate Dental Students at the University of Sharjah

**DOI:** 10.1055/s-0042-1743150

**Published:** 2022-03-13

**Authors:** Saaid Al Shehadat, Mohamed El-Kishawi, Asma AlMudalal, Asma AlSaqer, Aya Nassar, Leena Zihlif, Yazan Mahmoud, Venkateshbabu Nagendrababu, Thilla Sekar Vinothkumar

**Affiliations:** 1 Preventive and Restorative Dentistry, College of Dental Medicine, University of Sharjah, Sharjah, United Arab Emirates; 2 Restorative Dental Sciences, College of Dentistry, Jazan Universtiy, Jazan, Kingdom of Saudi Arabia

**Keywords:** iatrogenic errors, radiograph, root canal treatment, technical quality, undergraduate clinic, clinical audit, radiographic evaluation

## Abstract

**Objectives** The aim of this study was to determine the technical quality of root canal fillings and the presence of iatrogenic errors in the cases treated by undergraduate dental students using radiographic images.

**Materials and Methods** This study included 480 root-filled teeth, which were evaluated using intraoral periapical radiographic images. The technical quality of obturation was assessed by observing the length, density, and taperness of the root filling. Ledges, apical transportation, fractured instruments, zipping, and root perforation were recorded as iatrogenic errors. Teeth were classified as acceptable if the technical parameters were adequate and there were no iatrogenic errors.

**Statistical Analysis** Data were statistically analyzed using Pearson's chi-squared test.

**Results** The quality of root filling was acceptable in 183 of the 480 teeth. The rate of acceptable quality was higher for the teeth treated by 4th-year students (41.9%) than for those treated by 5th-year students (27.4%; *p* = 0.004). Length and taperness were adequate in more of the patients treated by the 4th-year students ( *p* <0.05). There was a significant difference in the incidence of ledge formation and apical transportation in relation to the student's level ( *p* <0.05).

**Conclusions** The technical quality of root fillings performed without iatrogenic errors by undergraduate dental students was declared acceptable in 38.1% of the assessed teeth. There was a noticeable difference in the quality of root canal treatment between the 4th- and 5th-year students.

**Clinical Relevance** The findings demonstrate that periodic assessment of the technical quality of root filling performed by undergraduate dental students provides feedback on curriculum standards.

## Introduction

Root canal treatment is an essential part of general dental practice. 1 2 The retention of natural permanent teeth is important for patients. Therefore, clinicians are expected to provide high-quality treatment. Undergraduate dental students should attain sound knowledge and adequate clinical skills in endodontics during their training. Dental school graduates should be able to competently perform root canal treatments. 2

The success of root canal treatment is dependent on having proper access cavity preparation, appropriate cleaning, shaping and filling of the root canal system, and maintaining adequate coronal and apical seal. 3 4 The poor technical quality of root fillings evaluated by radiographic images was related to posttreatment disease, which compromises the treatment outcome. 5 6 7 Many factors can affect the technical quality of root fillings, such as continuous tapering from the coronal part of the root canal to the apex, the absence of voids between the root filling and canal walls, and an optimal length of the root filling (i.e., 0.5 to 2 mm from the radiographic apex). 3 Unfortunately, the quality of root canal treatments performed by undergraduate dental students has been reported to be inadequate in many countries. 8 This finding could be attributed to inadequate undergraduate training. 9 In their clinical practice, general practitioners tend to follow the techniques learned during their undergraduate programs. 10 Hence, it is necessary to continuously assess the sequelae of clinical undergraduate training in dental schools.

In the College of Dental Medicine at the University of Sharjah, undergraduate dental students are taught endodontics in the 3rd, 4th, and 5th years of the Bachelor of Dental Surgery (BDS) program. In the 3rd year, students were trained to perform root canal treatment on extracted/typodont teeth in a preclinical laboratory. Students must complete root canal procedures in at least three anterior teeth, three premolars and three molars as preclinical exercises. In the 4th and 5th years, the students were trained in clinics, where they were expected to perform nonsurgical root canal treatment on both anterior and posterior teeth. Fourth-year students must complete root canal treatment on at least six teeth (including anterior and premolar teeth) to be eligible for the final examination. Fifth-year students must complete root canal treatment in at least three molars to be eligible for the final examination.

The aim of this study was to evaluate the technical quality of root fillings (length, dentistry, taperness) and to identify the presence of iatrogenic errors (ledge, apical transportation, fractured instrument, perforation, zipping) in patients treated by undergraduate dental students.

## Materials and Methods

Ethical approval of the current retrospective clinical audit was obtained from the Research Ethics Committee (REC-18–11–07–01-S). This audit randomly included the clinical records of patients (older than 16 years) who underwent root canal treatment and were treated by 4th-year (BDS 4) and 5th-year (BDS 5) clinical undergraduate dental students during the 2017–2018 academic year.

### Inclusion Criteria

The inclusion criteria were the following:

- Patients were included if their primary root canal treatment was performed in single or multirooted permanent teeth using a manual instrumentation technique, and obturation was performed by BDS 4 or BDS 5 students using a cold lateral compaction technique.
- Patients with at least three intraoral periapical (IOPA) radiographic images (preoperative, working length, and postoperative) were also included. The radiographic images showed the entire length of the root and at least 1 to 3 mm of the periapical area beyond the root apex.

### Exclusion Criteria

Patients with incomplete documentation, over-/underexposure to radiographic film, excessive shortening/elongation of their images, superimposition of root fillings, or adjacent anatomical structures were excluded from the study. Patients who underwent retreatment and those who were treated using rotary instruments were also excluded.

### Root Canal Treatment Protocol

All root canal treatments were performed under rubber dam isolation. Access cavity preparation was performed using round and Endo-Z access burs (Dentsply Maillefer, Ballaigues, Switzerland). The working length was determined using an apex locator (J Morita Root ZX II, USA) and confirmed using periapical radiographic images. Biomechanical root canal preparation was performed using a step-back technique, with stainless steel hand K files (Dentsply Maillefer, Ballaigues, Switzerland). The master apical file was two or three sizes larger than the initial binding file. Irrigation was achieved using 4% sodium hypochlorite solution (Vista Dental Products, Racine, Wisconsin, United States), using a side-vented needle–syringe combination. All teeth were obturated with gutta-percha (Dentsply Maillefer, Ballaigues, Switzerland) and AH plus sealer (Dentsply DeTrey, Konstanz, Germany) using the cold lateral compaction technique. Teeth were restored with glass ionomer temporary or composite resin permanent restorative materials. Finally, a postoperative periapical radiograph was taken to assess the obturation status. The clinics maintained an average staff to student ratio of 1:6.

### Assessing the Technical Quality of Root Fillings and Detecting Iatrogenic Errors

In the current study, the technical quality of the root fillings and iatrogenic errors were assessed using IOPA radiographic images. The technical quality of the root fillings (length, density, and taper) and iatrogenic errors (ledge formation, apical transportation, fractured instruments, zipping, root perforation) were assessed by modifying the criteria of Balto et al 5 and Barrieshi-Nusair et al, 9 respectively. The criteria for evaluating the quality of root canal filling are provided in Supplementary Table S1 (available in the online version only). The following criteria were used to identify the iatrogenic errors.

- Ledge formation: Indicated by incomplete root filling (i.e., at least 1 mm shorter than the working length and changes in the original root canal curvature).
- Apical transportation: The presence of filling material in the apical third outside the curve of the canal.
- Fractured instrument: A fractured instrument present inside the canal or partially outside the apical foramen.
- Zipping: the transportation of the apical termination of the filled canal in an elliptical shape.
- Root perforation: The presence of filling material in the periodontal space due to extrusion. It was subdivided based on the location of the root (i.e., coronal, middle, and apical). Strip and furcal perforations were considered to be coronal perforations. Apical perforation was diagnosed when the filling material was extruding through the apical foramen.

For a multirooted tooth, the quality of all canals was assessed simultaneously, and an overall score was provided. For multirooted teeth, each technical quality parameter was considered to be adequate only if every root filling was adequate. The tooth was declared to be acceptable in overall quality only if the length, density, and taperness were adequate and iatrogenic errors were absent. Image acquisition was performed for all patients, using a DIGORA Optime digital intraoral imaging plate system (DXR-50001; Soredex, Tuusula, Finland) with the exposure setting of 70 kV, 0.06 second, and the cone parallel to the radiographic plate. Two trained endodontists (SS, ME), who were trained in the quality assessment criteria, independently assessed the digital radiographic images, which were arranged in a PowerPoint file (Microsoft PowerPoint 2016, Microsoft, Redmond, Washington, United States). Any disagreement between the examiners was resolved by discussion.

### Statistical Analysis

Statistical analysis was performed using SPSS (Version 27, Chicago, Illinois, United States). Data on sex, student level, number of canals, tooth type, length, density, taperness, ledge, apical transportation, fractured instrument, perforation, and zipping were expressed as frequencies and percentages. Chi-squared tests were used to analyze the relationship between the independent variables (tooth type and student level) and the categorical variables (length, density, taperness) and procedural errors (ledge, apical transportation, broken files, perforation, and zipping). In addition, Cohen's kappa (κ) values for interexaminer agreement were analyzed using the data of all evaluated cases. Statistically significant levels were set at *p* < 0.05.

## Results

Of the 1,229 randomly screened patients, 414 were finally included based on the selection criteria. A total of 480 teeth (361 in males and 119 in females) were included in this study, with more patients treated by BDS 4 (74.2%) than by BDS 5 (25.8%; Table 1 ). The most frequently treated tooth type was maxillary premolars (31%), and the least frequently treated tooth was maxillary molars (6.3%). About half of the teeth in the study had single root canals (50.2%).

**Table 1:** Distribution of cases according to gender, student level, tooth type and number of root canals

Category	Frequency (%)
Gender	
Males	361 (75.2)
Females	119 (24.8)
Student level	
BDS 4	356 (74.2)
BDS 5	124 (25.8)
Tooth type	
Maxillary anteriors	118 (24.6)
Maxillary premolars	149 (31)
Maxillary molars	30 (6.3)
Mandibular anteriors	40 (8.3)
Mandibular premolars	70 (14.6)
Mandibular molars	73 (15.2)
Number of root canals	
1 canal	241 (50.2)
2 canals	146 (30.4)
3 canals	80 (16.7)
4 canals	13 (2.7)

**Fig. 1:**
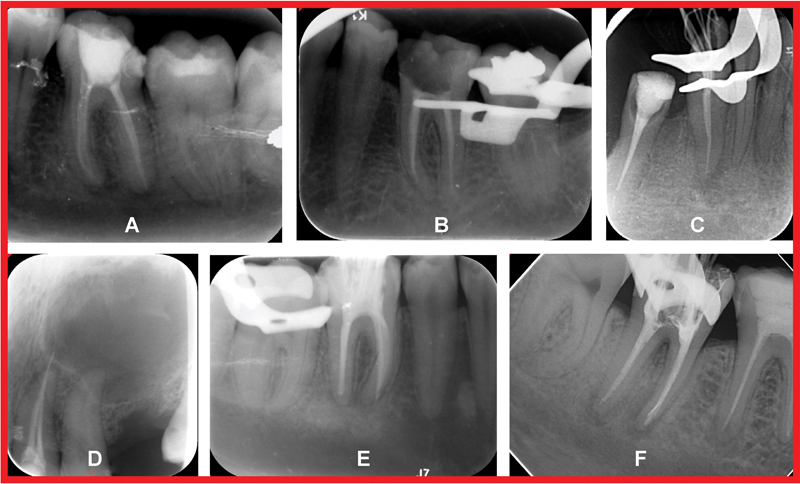
Samples of radiographic images used for the evaluation of the root canal fillings. ( **A** ) Adequate root filling. ( **B** ) Inadequate length. ( **C** ) Inadequate density and taperness. ( **D** ) Overfilled root canal with apical perforation. ( **E** ) Apical transportation in the mesial canals. ( **f** ) Separated instrument in the mesial canal.

The κ-values for interexaminer variability were 0.97, 0.92, and 0.95 for all lengths, densities, and taperness of filling, respectively. Among the iatrogenic errors, the κ-values were 0.88 (ledge), 0.82 (transportation), 0.96 (perforation), and 1 (fractured instruments). Fig. 1 shows samples of the radiographic images used for evaluating the quality of root canal fillings.

Table 2 shows that 183 (38.1%) teeth had acceptable overall quality. The maxillary anteriors had the highest overall quality (53.4%), followed by the mandibular premolars (42.9%), and the teeth with the lowest overall quality were the mandibular anteriors (22.5%) ( *p* < 0.001). In Table 3 , the overall quality of the teeth treated by BDS 4 (41.9%) was relatively better than that of BDS 5 (27.4%; *p* = 0.004). The length of the root canal filling was adequate in 73.5% of the teeth, underfilled in 18.1% and overfilled in 8.3% ( Table 4 ). The density and taperness were adequate in 57.7 and 66.3% of the teeth, respectively. Procedural errors (ledge, apical transportation, and fractured instrument) were found in 5.4, 3.5, and 1%, respectively ( Fig. 1 ). Perforation of the root canal was observed in the apical third (5.8%; Table 4 ). No cases of coronal and middle third perforations were reported. Zipping was not detected in any treated tooth.

**Table 2:** Overall quality of teeth with respect to tooth type

Tooth type	Number of teeth (%)	Quality (%)		*p* -Value a
		Acceptable	Not acceptable	
Maxillary				**<0.001**
Anteriors	118 (24.6)	63 (53.4)	55 (46.6)	
Premolars	149 (31)	56 (37.6)	93 (62.4)	
Molars	30 (6.3)	7 (23.3)	23 (76.7)	
Mandibular				
Anteriors	40 (8.3)	9 (22.5)	31 (77.5)	
Premolars	70 (14.6)	30 (42.9)	40 (57.1)	
Molars	73 (15.2)	18 (24.7)	55 (75.3)	
Total	480(100)	183 (38.1)	297 (61.9)	

a Pearson's chi-squared test; *p* > 0.05 is statistically significant.

**Table 3:** Overall quality of teeth with respect to student level

Student level	Number of teeth (%)	Quality (%)		*p* -Value a
		Acceptable	Not acceptable	
BDS4	356 (74.2)	149 (41.9)	207 (58.1)	0.004
BDS5	124 (25.9)	34 (27.4)	90 (72.6)	
Total	480(100)	183 (38.1)	297 (61.9)	

a Pearson's chi-squared test; *p* > 0.05 is statistically significant.

**Table 4:** Distribution of cases according to length, density, taperness, ledge, apical transportation, fractured instrument, perforation, and zipping

Category	Frequency (%)
Length	
Adequate	353 (73.5)
Underfilled	87 (18.1)
Overfilled	40 (8.3)
Density	
Adequate	277 (57.7)
Inadequate	203 (42.3)
Taperness	
Adequate	318 (66.3)
Inadequate	162 (33.8)
Ledge	
Present	26 (5.4)
Absent	454 (94.6)
Apical transportation	
Present	17 (3.5)
Absent	463 (96.5)
Fractured instrument	
Present	5 (1)
Absent	475 (99)
Perforation	
Coronal	0 (0)
Middle	0 (0)
Apical	28 (5.8)
Absent	452 (94.2)
Zipping	
Present	0 (0)
Absent	480 (100)

The chi-squared test showed statistically significant differences ( *p* < 0.05) in the length, density, and taperness of root filling among the different tooth types. The highest percentages of adequate root filling length, density, and taperness were for the maxillary anteriors ( *p* < 0.05; Table 5 ). There was significantly more apical transportation encountered in the mandibular molars than in the maxillary molars ( *p* <0.001). Apical perforation was found in 28 teeth, primarily in the maxillary premolars (10), followed by the mandibular molars (8). Apical transportation was significantly high in the mandibular molars ( *p* > 0.001; Table 6 ). The adequate length and taperness of the root fillings were significantly higher in the BDS 4 students than in the BDS 5 students ( *p* < 0.05). However, no difference was observed in the density of the root fillings ( Table 7 ). Compared with the BDS 4 students, apical transportation was found to be significantly higher in teeth treated by the BDS 5 students ( *p* < 0.0001; Table 8 ). The occurrence of ledge formation in patients treated by BDS 4 was greater than in patients treated by BDS 5 ( *p* < 0.05). Regarding perforation and fractured instruments, no differences were observed between the BDS 4 and BDS 5 students.

**Table 5:** Association between tooth type and technical quality aspects

Tooth type	Length (%)			*p* -Value a	Density (%)		*p* -Value a	Taperness (%)		*p* value a
	Adequate	Underfilled	Overfilled		Adequate	Inadequate		Adequate	Inadequate	
Maxillary				0.001			0.005			<0.0001
Anteriors	103 (87.3)	9 (7.6)	6 (5.1)		82 (69.5)	36 (30.5)		95 (80.5)	23 (19.5)	
Premolars	106 (71.1)	27 (18.1)	16 (10.7)		89 (46.2)	60 (4)		105 (70.5)	44 (29.5)	
Molars	18 (60)	9 (30)	3 (10)		14 (46.7)	16 (53.3)		15 (50)	15 (50)	
Mandibular										
Anteriors	26 (65)	12 (30)	2 (5)		15 (37.5)	25 (62.5)		17 (42.5)	23 (57.5)	
Premolars	57 (81.4)	10 (14.3)	3 (4.3)		35 (50)	35 (50)		46 (65.7)	24 (34.3)	
Molars	43 (58.9)	20 (28.6)	10 (13.7)		42 (57.5)	31 (42.5)		40 (54.8)	33 (45.2)	

a Pearson's chi-squared test; *p* > 0.05 is statistically significant.

**Table 6:** Association between tooth type and different iatrogenic errors

Tooth type	Ledge (%)		*p* value a	Apical transportation (%)		*p* value a	Fractured instrument (%)		*p* value a	Root perforation (%)		*p* value a
	Present	Absent		Present	Absent		Present	Absent		Present	Absent	
Maxillary			0.053			<0.001			0.297			0.08
Anteriors	1 (0.8)	117 (99.2)		0 (0.0)	118 (100)		0 (0.0)	118 (100)		5 (4.2)	113 (95.8)	
Premolars	7 (4.7)	142 (95.3)		0 (0.0)	149 (100)		2 (1.3)	147 (98.7)		10 (6.7)	139 (93.3)	
Molars	3 (10)	27 (90)		2 (6.7)	28 (93.3)		1 (3.3)	29 (96.6)		0 (0)	30 (100)	
Mandibular												
Anteriors	3 (7.5)	37 (92.5)		0 (0)	40 (100)		0 (0)	40 (100)		4 (10)	36 (90)	
Premolars	4 (5.7)	66 (94.3)		0 (0.0)	70 (100)		0 (0)	70 (100)		1 (1.4)	69 (98.6)	
Molars	8 (11)	65 (89)		15 (20.5)	58 (79.5)		2 (2.7)	71 (97.3)		8 (11)	65 (89)	

a Pearson's chi-squared test; *p* > 0.05 is statistically significant.

**Table 7:** Association between student level and technical quality

Student level	Length (%)			*p* value a	Density (%)		*p* value a	Taperness (%)		*p* value a
	Adequate	Underfilled	Overfilled		Adequate	Inadequate		Adequate	Inadequate	
BDS 4	277 (77.8)	51 (14.3)	28 (7.9)	0.001	213 (59.8)	143 (40.2)	0.111	245 (68.8)	111 (31.2)	0.044
BDS 5	76 (61.3)	36 (29)	12 (9.7)		64 (51.6)	60 (48.4)		73 (58.9)	51 (41.1)	

a Pearson's chi-squared test; *p* > 0.05 is statistically significant.

**Table 8:** Association between student level and iatrogenic errors

Student level	Ledge (%)		*p* value a	Apical transportation (%)		*p* value a	Fractured instrument (%)		*p* value a	Root perforation (%)		*p* value a
	Present	Absent		Present	Absent		Present	Absent		Present	Absent	
BDS 4	15 (4.2)	341 (95.8)	0.048	4 (1.1)	352 (98.9)	<0.0001	2 (0.6)	354 (99.4)	0.079	19 (5.3)	337 (94.7)	0.432
BDS 5	11 (8.9)	113 (91.1)		13 (10.5)	111 (89.5)		3 (2.4)	121 (97.5)		9 (7.3)	115 (92.7)	

a Pearson's chi-squared test; *p* > 0.05 is statistically significant.

## Discussion

The overall quality of root canal treatment is generally assessed retrospectively, based on the adequacy of obturation as well as the prevalence of iatrogenic errors. For all teeth in the current study, the technical outcome of root canal treatment performed by clinical undergraduate dental students was evaluated from the immediate postoperative IOPA radiograph(s).

Root filling quality was assessed using IOPA radiographic images. 11 Moreover, while assessing the quality, categorizing the teeth was difficult. 12 Thus, categorization was avoided in the current study. The lengths of root fillings determined by using three-dimensional limited field of view (FOV) cone beam computed tomography (CBCT) may not be identical to the lengths determined using IOPA radiographic images; the short fillings analyzed from IOPA radiographic images appeared as flush fillings in CBCT. 13 Although limited FOV CBCT has been recommended for predicting the factors affecting endodontic outcomes, 14 15 patients are routinely subjected to low-radiation postoperative IOPA radiograph unless otherwise indicated; therefore, FOV CBCT is hardly available in a retrospective audit.

Different criteria for the assessment of technical quality have been used in previous studies, some of which have evaluated quality based on the filling length, 16 length and lateral adaptation of the filling to the canal wall, 17 and length and density of the filling in the apical third of root canal. 1 18 However, the method of radiographic projection affects the length and density in the radiograph. The length of root fillings in IOPA radiographic images is usually predicted to be shorter with the bisecting-angle technique than with the parallel technique. 19 Hence, few studies 5 9 20 have focused on the taperness of the root filling in addition to length and density to fulfil the mechanical, biologic, and technical objectives of endodontic treatment. 21 22 Accordingly, we assessed all three parameters (length, density, and taperness) in the current study.

In addition to optimal cleaning and shaping, the technical conditions of obturation play a vital role in favorable endodontic outcomes. 22 Many studies have evaluated root filling quality based on technical aspects alone. 9 18 20 23 In contrast, other studies have incorporated the “identification of iatrogenic errors” as an important criterion in their methodologies. 5 24 25 Although the technical quality of the root filling (i.e., length, density, and taperness) is a significant parameter, the absence of errors corresponds to the clinical skills of the students and the integrity of preclinical and clinical training provided to them.

Segura-Egea et al 17 evaluated root filling length based on the criterion of being 3 mm from the radiographic apex. However, the criterion of ≤2 mm has been considered as the gold standard in most of the studies 18 20 21 ; thus, that criterion was applied in the present study. Regarding the density of the root filling, the presence of voids was recorded as being inadequate in the present study to ensure optimum condensation and close adaptation of material to the canal walls. The reproducibility of such voids in radiographic images is challenging, and it depends on the angle of the radiographic projection. 26

The filling quality of single-rooted teeth (anteriors and premolars) has been reported by Lynch and Burke to be acceptable at up to 70%. 23 Most of these teeth possess invariably straight canals. Nevertheless, canal curvature is a significant challenging factor for the students; 58.4% of all teeth and 61% of molars with severely curved canals were associated with ledges. 25 Therefore, the adequacy of student training and skills is perfectly judged by assessing the quality of the endodontic treatment in both anterior and posterior teeth, with varying grades of difficulty. 27

In the present study, the incidence of adequate length was 73.5%, which is comparable to the result of Turkish students (69.6%), as reported by Er et al. 28 In contrast, studies from Taiwan, Saudi Arabia, Greece, and the United Kingdom reported results ranging from 27 to 67%. 1 5 25 29 The percentage variations could be attributed to differences in the radiographic projection techniques of undergraduate dental students as well as the use of electronic apex locators. Compared with density and taperness, root filling length is a relatively reproducible quality parameter. 25 26 The root filling density depends on the radiographic projection and radiopacity of the material used. 18 The density was adequate in 57.7% of the cases in the current study, which is superior to previous reports of 35% 5 and 42.7%. 18 These values are not comparable due to sample size variation. The adequacy percentages of length, density and taperness were higher for the maxillary anteriors and maxillary premolars. This result could be attributed to the relatively high number of cases recruited. According to the European Society of Endodontology, it is mandatory to prepare root canals tapering from the crown to the apex while maintaining apical constriction. 3 In the present study, the proportion of teeth with adequate taperness (66.3%) was higher compared with a study of Saudi students (60%) 5 but lower compared with a study of students from Jordan and Turkey. 9 28 Nevertheless, visual assessment of taper from radiographic images is arbitrary and highly subjective. 5

The most commonly observed iatrogenic error was apical perforation (5.8%), followed by ledges (5.4%), apical transportation (3.5%), and fractured instruments (1%). Ledges and apical transportation were predominant in mandibular molars. This finding could be due to the variations in the root curvature, inflexibility of the hand files used, and complexity of the root canal system. 30 However, the observed frequencies were relatively lower than those of earlier studies. 5 25 There is a possibility of missing iatrogenic error data, which may be due to the following: (1) limited visibility of perforation, zipping, and ledges in radiographic images, depending upon the three-dimensional location of errors over the root surface and (2) root curvature in the buccolingual orientation in relation to the direction of radiographic projection. 31 Interestingly, ledges were more frequently encountered in cases treated by BDS 5 students than in those treated by BDS 4 because the number of cases was relatively lower for BDS 5. In addition, compared with the BDS 4 group, the BDS 5 group was encouraged to treat a greater number of molars with difficult canal curvature. Therefore, when interpreting the results, it must be considered that root canal treatment was performed by students with limited endodontic experience. 32

In general, 38.1% of the teeth treated by BDS 4 and BDS 5 students together met the requirements of acceptable overall quality, which is higher than the 23% reported by Balto et al 5 but close to the 39% reported by Dugas et al. 33 However, the obtained value is lower than 47% 9 and 55%. 25 These studies are not truly comparable due to variations in the study design and assessment criteria. 9 Undoubtedly, the maxillary anteriors with simple root canal anatomy received more root fillings (53.4%) with acceptable overall quality. The BDS 4 students achieved a more acceptable root filling quality (41.9%) than the BDS 5 students (27.4%), which contradicts the premise that the treatment quality of students improves with experience. This result could reflect certain deficiencies and indicate the need for improvement of the school's endodontic curriculum. 9 This improvement includes the use of simulated plastic teeth at the early stages of learning and designing endodontic learning tasks from simple to complex starting with single straight canals and progressing to multiple curved canals to minimize anatomical variation and complexity of the root canal system. 30

### Strength and Limitations

While acknowledging that filling in each root contributes to the treatment outcomes of multirooted teeth, strict adherence to the criteria of excluding teeth with either inadequate technical parameters or iatrogenic errors ensured the accuracy of the overall quality results in the current study. The κ-values were greater than 0.81 for all the evaluated criteria, which demonstrates the existence of excellent interexaminer agreement. This finding could be attributed to the adequate calibration of examiners and distinct assessment criteria. However, radiographic images are two-dimensional, and the use of more than one IOPA radiograph provides valuable information, such as multiple canals, voids, adaptation of filling material to the canal wall, and anatomic structures. 25 An orthoradial image alone is inaccurate in the assessment of voids, even for single canal teeth. 26 Moreover, it is often difficult to detect zipping in IOPA radiographic images.

## Conclusion

The overall acceptable quality of root canal treatment delivered by undergraduate dental students without iatrogenic errors was 38.1%. The frequency of acceptable root fillings was higher in maxillary anteriors with simple root canal anatomy. Treatments completed by the 4th-year students were more acceptable than those completed by the 5th-year students. It is recommended that such retrospective audits should be performed regularly to improve the standards of patient care delivered during undergraduate clinical dental courses.
