# Supplementary material for: An Audit of the Technical Quality and Iatrogenic Errors of Root Canal Treatment by Undergraduate Dental Students at the University of Sharjah
Source: Eur J Dent. 2022 Mar 13;17(1):191–9. doi: 10.1055/s-0042-1743150 (PMC9949975; doi:10.1055/s-0042-1743150)
Supplement: Supplementary file 1 — Supplementary Material [file 10-1055-s-0042-1743150-s21121887.pdf]

Supplementary Table S1 Criteria of radiographic interpretation for the technical quality of root canal treatment

Variable	Criteria	Definition
Length of root canal filling	Adequate	Root filling ends at 0–2 mm of radiographic apex
	Underfilled	Root filling ending >2 mm short of radiographic apex
	Overfilled	Root filling ending beyond the apex
Density of root canal filling	Adequate	Uniform density of root filling without voids or space
	Inadequate	No uniform density of root filling with clear space is visible
Taper of root canal filling	Adequate	Consistent taper from the coronal to the apical part
	Inadequate	No consistent taper from coronal to apical part

 © 2022. The Author(s).
